# Lifestyle and metabolic factors in relation to shoulder pain and rotator cuff tendinitis: A population-based study

**DOI:** 10.1186/1471-2474-11-165

**Published:** 2010-07-20

**Authors:** Martti Rechardt, Rahman Shiri, Jaro Karppinen, Antti Jula, Markku Heliövaara, Eira Viikari-Juntura

**Affiliations:** 1 Centre of Expertise for Health and Work Ability, Finnish Institute of Occupational Health, Finland; 2 Department of Physical Medicine and Rehabilitation, University of Oulu, Finland; 3 Department of Health and Functional Capacity, National Institute for Health and Welfare, Helsinki and Turku, Finland

## Abstract

**Background:**

Shoulder pain is a common health problem. The purpose of this study was to assess the associations of lifestyle factors, metabolic factors and carotid intima-media thickness with shoulder pain and chronic (> 3 months) rotator cuff tendinitis.

**Methods:**

In this cross-sectional study, the target population consisted of subjects aged 30 years or older participating in a national Finnish Health Survey during 2000-2001. Of the 7,977 eligible subjects, 6,237 (78.2%) participated in a structured interview and clinical examination. Chronic rotator cuff tendinitis was diagnosed clinically. Weight-related factors, C-reactive protein and carotid intima-media thickness were measured.

**Results:**

The prevalence of shoulder joint pain during the preceding 30 days was 16% and that of chronic rotator cuff tendinitis 2.8%. Smoking, waist circumference and waist-to-hip ratio were related to an increased prevalence of shoulder pain in both genders. Metabolic syndrome, type 2 diabetes mellitus and carotid intima-media thickness were associated with shoulder pain in men, whereas high level of C-reactive protein was associated with shoulder pain in women. Increased waist circumference and type 1 diabetes mellitus were associated with chronic rotator cuff tendinitis in men.

**Conclusions:**

Our findings showed associations of abdominal obesity, some other metabolic factors and carotid intima-media thickness with shoulder pain. Disturbed glucose metabolism and atherosclerosis may be underlying mechanisms, although not fully supported by the findings of this study. Prospective studies are needed to further investigate the role of lifestyle and metabolic factors in shoulder disorders.

## Background

Shoulder pain is a common clinical symptom and a notable cause of work disability and health care costs [1]. In general populations, the prevalence of shoulder pain during the preceding 30 days ranges between 18% and 31% [2]. Shoulder structures are liable to traumas in accidental injuries such as falls. Moreover, the rotator cuff tendons undergo degenerative changes with age, predisposing to tendinosis and associated conditions. Shoulder pain may also reflect shoulder joint disorders such as adhesive capsulitis, synovitis, glenohumeral instability [3], as well as, particularly in aging people, acromioclavicular and glenohumeral osteoarthritis.

Shoulder pain and rotator cuff disorders may be caused or aggravated by a range of environmental and individual factors. Physical load factors at work have shown an association with both shoulder pain and disorders [4-6]. A number of studies suggest a link between shoulder disorders and metabolic factors, such as obesity [7,8] and diabetes mellitus [8]. Previous studies, however, included mostly selected populations and limited information about metabolic factors. Moreover, inconsistent findings have been reported regarding the role of smoking and physical exercise [8].

The metabolic syndrome, an increasing health problem in industrialised countries, involves central obesity, dyslipidemia and insulin resistance, and increases the risk of cardiovascular disease [9]. C-reactive protein (CRP) and increased carotid artery intima-media thickness (IMT) are risk indicators of atherosclerotic vascular diseases. Previous studies have also shown an association between CRP and upper extremity osteoarthritis [10,11]. However, we are not aware of studies on the relations of CRP, the metabolic syndrome, and carotid IMT with shoulder pain and rotator cuff tendinitis.

We hypothesised that lifestyle factors, metabolic factors and carotid IMT are associated with shoulder pain and rotator cuff tendinitis. To test this hypothesis, we carried out a cross-sectional health survey in the general Finnish adult population.

## Methods

### Population

The target population of this cross-sectional study comprised of men and women aged 30 years or over residing in Finland between the fall of 2000 and the spring of 2001. A two-stage stratified cluster design was used to obtain a representative sample of the Finnish population, first stratified into 5 university hospital regions each containing roughly 1 million inhabitants. From each university hospital region, 16 health care districts were sampled as clusters (altogether 80 health care districts in the whole country). The 15 largest health centre districts in the country were all selected in the sample with probability 1, and the remaining 65 health centre districts were selected by systematic probability proportional to size (PPS) sampling in each stratum. Thus the 80 health centre districts were the primary sampling units. The ultimate sampling units were persons who were selected by systematic sampling from the health centre districts. Persons aged 80 years or over were oversampled by doubling the sampling fraction. For the 15 largest health centre districts, the sample sizes were proportional to population size. In the 65 PPS sampled clusters the sample sizes were equal within each university hospital region so that the total number of persons drawn from a university hospital region was proportional to the corresponding population size [12].

**Figure 1:**
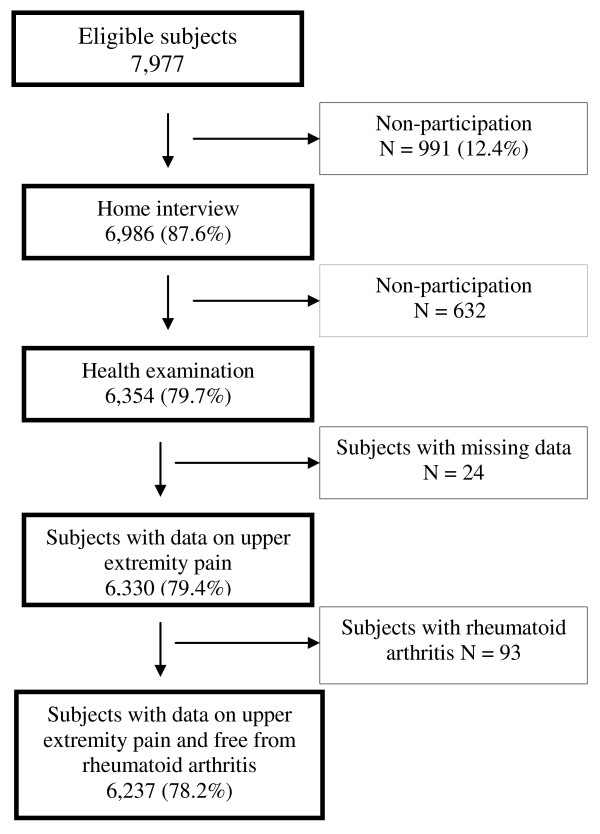
**Flow chart of the study population, Health 2000 Survey, 2000-2001**.

The original sample of the Health 2000 survey consisted of 8,028 subjects aged 30 years or over. Of them 51 were deceased before interview, 6,986 (87.6%) were interviewed and 6,354 (79.7%) participated in the health examination (Figure 1). Subjects with missing information on shoulder disorders (N = 24) and those with clinically diagnosed rheumatoid arthritis and a positive rheumatoid factor (N = 93) were excluded, leaving 6,237 (78.2%) qualified subjects.

The Ethical Committee of Epidemiology and National Welfare of Helsinki University Hospital District approved the Health 2000 survey on the 21^st ^of September 1999. All participants gave an informed consent.

### Outcomes

All subjects were examined with a structured protocol that included a symptom interview on musculoskeletal complaints by a trained nurse and a standardized physical examination by a trained physician. Joint symptoms were first inquired with a general question "Have you had joint pain, ache or motion tenderness in one or more joints during the preceding 30 days?" After this the examinee was shown a manikin, in which s/he was asked to point out the painful joint regions.

In addition to shoulder joint pain using a manikin, the examination included also separate questions on pain perceived in the neck, shoulder, or neck-shoulder angle during the preceding 30 days. In the present study we included only shoulder pain. We defined unilateral shoulder joint pain as having pain either in right or left shoulder during the preceding 30 days, and bilateral shoulder joint pain as having pain in both shoulders during the preceding 30 days. Chronic rotator cuff tendinitis (tendinopathy) was defined as history of pain in the rotator cuff region (i.e. the deltoid or the epaulette region) lasting for at least 3 months, plus pain during the past month preceding the examination, and pain in one or more active resisted movements (abduction, external rotation, internal rotation) and/or painful arc of shoulder abduction. Diagnostic certainty was classified into two levels (possible or definite) according to a physician's assessment; however, the categories were combined in the analysis.

### Quality assessment

Two pilot studies were carried out 7 and 3 months before the field work started, in order to test and improve the methods. Detailed written instructions for the assessment of physical status were provided before the field examination. All staff members, including physicians, attended a 3-week training course. Other quality control measures included observation, video recording with feedback on examination technique, and repeated and parallel measurements.

### Independent variables

Information on sociodemographic factors, lifestyle factors and work related physical load factors was obtained with interviews http://www.terveys2000.fi/indexe.html[12]. The subjects were defined as 1) current smokers, if they smoked cigarettes, cigars or a pipe at the time of interview; 2) former smokers if they had smoked at least for one year in the past and were not current smokers; 3) occasional smokers; and 4) never smokers. For current smokers pack years were estimated and grouped into three levels (< 10, 10-20, > 20). Leisure time physical exercise (duration of at least 30 minutes causing sweating and breathlessness) was assessed by a single global question and classified into one of three groups: ≤ 1, 2-3, and ≥ 4 times per week.

Weekly consumption of alcohol was recorded in units (drinks, serving portions) and converted into grams of absolute alcohol. Alcohol consumption was grouped into 4 levels, none (0 grams of alcohol), light, moderate, or heavy. We defined the three latter categories using distribution based cut points. We classified alcohol consumers into three equal-sized groups.

We collected information on current exposure to occupational physical load factors, such as working with hands above the shoulder level for at least one hour per day, manual handling of loads heavier than 5 kg at least two times per minute for a minimum of two hours daily and manual handling of loads heavier than 20 kg for at least 10 times per day, working with a vibrating tool for at least two hours, work demanding high handgrip forces for at least one hour, and repetitive movements of the hands or wrists for at least two hours [13].

Height, weight, waist circumference and hip circumference were measured. Waist circumference measurement was taken half way between the lowest rib and the iliac crest. Body mass index (BMI) was classified into underweight (BMI < 18.5), normal weight (BMI 18.5-24.9), overweight (BMI 25.0-29.9) and obesity (BMI ≥ 30.0). Waist circumference was grouped into three levels; in men < 94.0 cm, 94.0-101.9 cm, ≥ 102.0 cm and in women < 80.0 cm, 80.0-87.9 cm and ≥ 88.0 cm [14]. Waist-to-hip ratio was classified into three groups: in men < 0.9, 0.9-1.0, > 1.0 and in women < 0.8, 0.8-0.9 and > 0.9 [15].

The diagnosis of diabetes mellitus was based on fasting blood glucose ≤7.0 mmol/l (126 mg/dL) [16], and/or a known previous diagnosis of diabetes mellitus, or glucose lowering medication. For the analysis of serum glucose, insulin, high-density lipoprotein (HDL) cholesterol, triglycerides and highly sensitised C-reactive protein, the participants donated fasting blood samples, which were centrifuged and placed into deep freezers at -20°C and within one week transferred and stored in deep freezers at -70°C for analyses, using commercial enzymatic methods and clinical chemistry autoanalysers, at the National Public Health Institute's laboratory. We defined the metabolic syndrome according to the criteria of the American Association of Clinical Endocrinologists [17] when at least 3 of the following criteria were present: 1) Central obesity, defined as waist circumference > 102 cm in men and > 88 cm in women; 2) high fasting triglycerides, defined as ≥ 1.7 mmol/l (> 150 mg/dL); 3) low HDL cholesterol defined as < 1.0 mmol/l (< 40 mg/dL) in men and < 1.3 mmol/l (< 50 mg/dL) in women ; 4) elevated blood pressure, defined as systolic blood pressure ≥ 130 mm Hg or diastolic blood pressure ≥ 85 mm Hg; and 5) impaired fasting glucose, defined as fasting glucose ≥ 6.1 mmol/l (110 mg/dL). The homeostasis model assessment (HOMA) of insulin resistance was defined as serum insulin pmol/l × glucose mmol/l/22.5 [18]. High serum high-sensitive C-reactive protein (hs-CRP) was defined as ≥ 3 mg/l (0.3 mg/dL)[19].

### Carotid artery intima-media thickness

Earlier, we described the details of ultrasound measures of the carotid artery IMT [20]. Ultrasound measures of carotid IMT were performed in a sub-sample of subjects aged 45 to 74 who resided within 200 kilometres from the six study clinics. These were located in four hospitals and two research institutions which all had cardiovascular ultrasound equipment with a linear array transducer available. The six study clinics cover six Finnish towns and their surrounding areas located in the southern (Helsinki), southwestern (Turku), middle (Tampere), eastern (Kuopio and Joensuu), and northern (Oulu) parts of the country. Subjects (N = 1867) who fulfilled these eligibility criteria were invited and 1526 (82%) of them participated in the carotid artery ultrasound study. We assessed the relation between IMT and shoulder disorders among 1353 (72%) subjects who had data on both carotid artery ultrasound and shoulder disorders available. In our previous report [20] we compared the characteristics of the study population with those of original Health 2000 sample. The study population had slightly lower BMI and higher education than the original Health 2000 population; otherwise the study population was well representative of the original Health 2000 population.

A high-resolution B-mode carotid ultrasound examination of the carotid artery was performed first on the distal 1 cm of the common carotid artery and then on the carotid artery bulb. The IMT was measured from three digitized end diastole images of the common carotid artery (lateral angle) and the carotid bulb (three interrogation angles). In the analysis of this study we used an average of these six measures.

### Statistical analysis

Statistical significance (*P *< 0.05, two-tailed) for differences in background characteristics between men and women was assessed by chi-squared test for the categorical variables and by two-sample t-test for the continuous variables. We ran logistic regression models to study the determinants of shoulder joint pain and chronic rotator cuff tendinitis. We used two outcomes; shoulder joint pain for at least a day during the preceding 30 days (no, unilateral, bilateral) and chronic rotator cuff tendinitis (no, yes). For shoulder joint pain we performed multinomial logistic regression models. We hypothesized that the associations of lifestyle and metabolic factors with shoulder pain would be stronger for bilateral than unilateral shoulder pain via a systemic effect. We therefore performed separate analyses for unilateral and bilateral shoulder joint pain using multinomial logistic regression. There were interactions between lifestyle factors and gender, and between carotid IMT and gender for shoulder joint pain. We therefore used gender-specific multivariable models. The associations of lifestyle factors, metabolic factors and carotid IMT with shoulder pain and chronic rotator cuff tendinitis were controlled for age (continuous), years of education (continuous) and physical work load factors (categorical). These factors were associated with shoulder pain and chronic rotator cuff tendinitis in univariable analyses. Population weighting was used in estimating the prevalence, confidence intervals (CI), and odds ratios (OR) to correct age, sex, residential district, and language distributions of the study sample to correspond to those of the general Finnish population. Stata, version 10, software was used to conduct survey analyses.

## Results

### Background Characteristics

Women were on average 2 years older than men (Table 1). Men had higher body mass index and higher waist circumference compared to women; however, women had more abdominal obesity compared with the reference values. Men also had higher insulin resistance, thicker carotid intima-media and were more frequently current smokers than women. Type 1 diabetes mellitus was more common in men than in women. Women reported being more active during leisure time than men. There were no differences between the genders regarding years of education, type 2 diabetes mellitus or the prevalence of shoulder joint pain and chronic rotator cuff tendinitis.

**Table 1:** Background characteristics of the study subjects free of rheumatoid arthritis (N = 6,237), weighted proportion or mean and 95% confidence intervals, Health 2000 Survey, 2000-2001

Characteristic	Men (N = 2850)			Women (N = 3387)			P-value
			
	%	Mean	95% CI	%	Mean	95% CI	
Mean age		50.8	50.2-51.3		52.9	52.4-53.4	< 0.001
							
Years of education		11.3	11.1-11.4		11.4	11.2-11.5	0.36
							
Body mass index (kg/m^2^)		26.6	26.4-26.8		25.9	25.7-26.0	< 0.001
							
Waist circumference (cm)		97.7	97.3-98.2		88.4	87.9-88.9	< 0.001
							
Insulin resistance^1 ^(HOMA-IR, mean)		2.8	2.5-3.1		2.3	2.1-2.4	0.001
							
Current smoking	32		30-34	20		18-22	< 0.001
							
Exercise (times/week)							
. ≤ 1	43		41-45	39		38-41	
2-3	32		30-34	33		31-35	
. ≥ 4	25		24-27	28		26-30	0.01
							
Metabolic syndrome^2^	30		28-32	31		29-33	0.56
							
Diabetes							
Type 1	0.8		0.5-1.1	0.4		0.2-0.7	
Type 2	5		4-6	5		4-6	0.24
							
High C-reactive protein (> 3 mg/L)	16		14-17	18		16-19	0.03
							
Carotid intima-media thickness		0.97	0.95-0.99		0.90	0.88-0.92	< 0.001
							
Shoulder joint pain							
Unilateral^3^	9		8-11	10		8-12	
Bilateral^4^	6		4-7	7		6-8	0.03
							
Chronic rotator cuff tendinitis^5^							
Possible	1.6		1.1-2.3	1.6		1.2-2.1	0.99
Probable	1.2		0.8-1.6	1.2		0.9-1.7	0.81

^1 ^HOMA-IR: Serum insulin pmol/l × glucose mmol/l/22.5

^2 ^Three of the following criteria present: 1) Central obesity, defined as waist circumference > 102 cm in men and > 88 cm in women; 2) triglycerides ≥ 1.7 mmol/l; 3) HDL < 1.0 mmol/l in men and < 1.3 mmol/l in women; 4) systolic blood pressure ≥ 130 mm Hg or diastolic blood pressure ≥ 85 mm Hg; and 5) fasting glucose ≥ 6.1 mmol/l.

^3 ^Pain either in right or left shoulder during the preceding 30 days.

^4 ^Pain in both shoulders during the preceding 30 days.

^5 ^History of pain in the rotator cuff region lasting for at least 3 months, plus pain during the past month preceding the examination, moderately or slightly restricted movement in the shoulder joint, and pain in one or more active resisted movements (abduction, external rotation, internal rotation), or painful arc of shoulder abduction.

### Shoulder Joint Pain

In univariable analyses (Additional file 1: appendix 1), age, education, BMI, waist circumference, waist-to-hip ratio, metabolic syndrome, diabetes and physical load factors were associated with shoulder joint pain in both men and women, while smoking and carotid IMT were associated only in men, and insulin resistance and CRP only in women.

After adjustment for age, education and occupational physical load factors, current smoking was associated with unilateral shoulder pain in men but not in women (Table 2). Currently smoking men who had smoked for more than 20 pack-years were at highest risk of unilateral shoulder pain. Currently smoking women who had smoked for 10-20 pack-years were at highest risk of bilateral shoulder pain. Physical activity was not significantly associated with shoulder pain in either gender. Shoulder pain was associated with body mass index, waist circumference and waist-to-hip ratio in both women and men. The association between waist circumference and shoulder pain did not change after further adjustment for height. The associations were stronger for unilateral shoulder pain in men and for bilateral shoulder pain in women. Metabolic syndrome and type 2 diabetes mellitus were associated with unilateral shoulder pain in men. High CRP was associated with bilateral shoulder pain in women. Alcohol consumption was not related to shoulder joint pain in either gender (data not shown).

**Table 2:** Gender-specific odds ratios of unilateral or bilateral shoulder pain by lifestyle and metabolic factors, Health 2000 Survey, 2000-2001

Characteristic	Men							Women						
		
	Sample	Unilateral^4^			Bilateral^5^			Sample	Unilateral^4^			Bilateral^5^		
				
		% of outcome	OR	95% CI	% of outcome	OR	95% CI		% of outcome	OR	95% CI	% of outcome	OR	95% CI
Smoking status														
Never smoker	526	6.6	1		6.0	1		1487	11.6	1		8.6	1	
Former smoker	1005	10.2	1.4	0.9-2.1	7.1	0.9	0.6-1.5	612	9.5	1.0	0.7-1.3	6.6	1.1	0.7-1.6
Occasional smoker	168	7.8	1.3	0.6-2.4	3.1	0.7	0.3-1.8	156	8.1	1.0	0.5-2.0	1.4	0.3	0.1-1.5
Current smoker														
< 10 pack-years	255	10.3	1.5	0.9-2.4	5.3	0.9	0.4-1.7	218	9.8	1.1	0.7-1.9	3.3	0.7	0.3-1.7
10-20 pack-years	152	7.6	1.2	0.5-2.4	2.6	0.5	0.1-1.9	175	7.1	0.8	0.4-1.7	8.7	1.8	1.0-3.1
> 20 pack-years	349	13.6	1.9	1.3-2.9	4.6	0.7	0.4-1.4	162	6.3	0.6	0.3-1.2	4.0	0.6	0.3-1.4
														
Exercise (times/week)														
≥ 1	1170	8.8	1		5.4	1		1281	9.6	1		8.0	1	
2-3	879	11.1	1.3	0.9-1.8	4.8	1.0	0.6-1.5	1050	10.6	1.1	0.8-1.5	5.9	0.8	0.5-1.1
≤ 4	697	7.0	0.7	0.5-1.1	6.6	1.0	0.6-1.6	893	9.5	0.9	0.6-1.2	6.2	0.7	0.4-1.0
														
Body mass index														
Normal (18.5-24.9)	967	7.7	1		4.3	1		1489	8.2	1		5.1	1	
Underweight (< 18.5)	13	0	-	-	0	-	-	46	3.7	0.4	0.1-2.0	2.3	0.4	0.1-3.5
Overweight (25.0-29.9)	1231	9.6	1.3	0.9-1.7	6.2	1.4	0.9-2.1	1011	11.6	1.2	0.9-1.6	7.1	1.1	0.7-1.5
Obese (≥ 30.0)	455	11.5	1.4	1.0-2.0	6.4	1.4	0.8-2.2	529	11.8	1.3	0.9-1.8	10.3	1.6	1.0-2.4
														
Waist circumference														
Men: < 94.0 cm, Women: < 80 cm	1053	6.1	1		4.7	1		918	6.5	1		2.8	1	
Men: 94.0-101.9 cm	802	10.9	1.8	1.2-2.5	5.5	1.1	0.7-1.7	798	10.5	1.5	1.1-2.1	6.2	1.9	1.2-3.2
Women: 80.0-87.9 cm														
Men: ≥ 102.0 cm	933	11.5	1.8	1.3-2.5	6.6	1.2	0.8-1.7	1580	11.3	1.4	0.99-2.0	10.1	2.5	1.6-4.0
Women: ≥ 88.0 cm														
														
Waist-to-hip ratio^1^														
Normal	326	4.3	1		1.8	1		606	8.7	1		4.0	1	
Increased	1584	9.3	2.1	1.2-3.7	5.9	2.5	1.1-5.9	1858	9.0	0.9	0.6-1.2	5.8	1.0	0.6-1.6
High	878	11.2	2.4	1.3-4.4	6.4	2.4	1.0-5.8	831	12.3	1.0	0.7-1.5	12.3	1.8	1.1-3.1
														
Metabolic syndrome^2^														
No	1945	8.0	1		5.2	1		2273	9.4	1		5.5	1	
Yes	842	12.5	1.5	1.2-1.9	6.2	1.0	0.7-1.5	1033	10.7	0.8	0.6-1.1	10.8	1.2	0.9-1.7
														
Insulin resistance^3^														
Mean, per each standard deviation increase	2792		1.0	0.9-1.1		1.0	0.9-1.1	3318		1.1	0.9-1.3		1.1	0.9-1.3
														
Diabetes														
No	2637	9.0	1		5.1	1		3135	9.5	1		6.7	1	
Type 1	21	0	-		10.0	2.2	0.4-11.1	13	0	-		15.4	1.5	0.3-7.4
Type 2	144	16.1	1.7	1.0-2.9	13.3	1.9	0.98-3.6	177	16.3	1.3	0.8-1.9	13.8	1.3	0.7-2.2
														
C-reactive protein														
Low ≤ 3 mg/L	2339	8.8	1		5.4	1		2697	9.7	1		6.4	1	
High > 3 mg/L	436	11.8	1.3	0.9-1.9	6.8	1.1	0.7-1.8	582	10.9	1.0	0.7-1.4	10.6	1.4	1.0-2.1
														
Carotid intima-media thickness														
Mean IMT, per each standard deviation (0.23 mm) increase	599		1.2	0.9-1.6		1.3	0.9-1.7	723		0.7	0.4-1.0		0.9	0.6-1.3

Odds ratios were adjusted for age, education and physical work load factors (working with hands above the shoulder level for at least one hour per day, manual handling of loads heavier than 5 kg at least two times per minute for a minimum of two hours daily and manual handling of loads heavier than 20 kg for at least 10 times per day, working with a vibrating tool for at least two hours, work demanding high handgrip forces for at least one hour, and repetitive movements of the hands or wrists for at least two hours).

^1 ^In men: Normal < 0.9, Increased 0.9-1.0 and High > 1.0. In women: Normal < 0.8, Increased 0.8-0.9 and High > 0.9.

^2 ^Three of the following criteria present: 1) Central obesity, defined as waist circumference > 102 cm in men and > 88 cm in women; 2) triglycerides ≥ 1.7 mmol/l; 3) HDL < 1.0 mmol/l in men and < 1.3 mmol/l in women; 4) systolic blood pressure ≥ 130 mm Hg or diastolic blood pressure ≥ 85 mm Hg; and 5) fasting glucose ≥ 6.1 mmol/l.

^3 ^HOMA-IR: Serum insulin pmol/l × glucose mmol/l/22.5

^4 ^Pain either in right or left shoulder during the preceding 30 days.

^5 ^Pain in both shoulders during the preceding 30 days.

In men, the prevalence of shoulder pain increased non-significantly proportional to increasing carotid intima-media thickness (Table 2). However, the association between carotid IMT and shoulder pain was statistically significant in men aged 60 or older: The odds ratio of unilateral or bilateral shoulder pain for each standard deviation increase in carotid IMT was 1.4 (95% CI 1.0-1.9).

### Chronic Rotator Cuff Tendinitis

In univariable analyses (Additional file 1: appendix 2), age, education and waist circumference were associated with chronic rotator cuff tendinitis in both men and women, while waist-to-hip ratio and diabetes were associated only in men.

Smoking and physical exercise were not associated with chronic rotator cuff tendinitis after adjustment for age, education and occupational physical load factors (Table 3). Overweight and obese men had a high prevalence of chronic rotator cuff tendinitis; however a statistically significantly increased risk was observed only for increased waist circumference. In women weight-related factors were not statistically significantly associated with chronic rotator cuff tendinitis. However, the odds ratios were above unity for those with increased waist circumference. Type 1 diabetes mellitus was associated with chronic rotator cuff tendinitis in men. Metabolic syndrome, insulin resistance, CRP, alcohol consumption and carotid IMT were not associated with chronic rotator cuff tendinitis in either men or women.

**Table 3:** Gender-specific odds ratios of chronic rotator cuff tendinitis by lifestyle and metabolic factors, Health 2000 Survey, 2000-2001

Characteristic	Men				Women			
		
	Sample	% of outcome	OR	95% CI	Sample	% of outcome	OR	95% CI
Smoking status								
Never smoker	537	2.4	1		1497	3.5	1	
Former smoker	1014	3.5	1.3	0.7-2.3	621	3.1	1.0	0.5-1.8
Occasional smoker	171	1.2	0.6	0.1-2.5	160	0.7	0.3	0.1-2.0
Current smoker								
< 10 pack-years	253	0.8	0.3	0.1-1.6	219	2.4	0.9	0.3-2.4
10-20 pack-years	154	0.6	0.3	0.1-2.3	178	3.6	1.4	0.6-3.2
> 20 pack-years	351	3.4	1.3	0.5-3.3	162	1.2	0.4	0.1-1.7
								
Exercise (times/week)								
. ≤1	1183	2.2	1		1289	2.5	1	
2-3	886	3.3	1.6	0.9-2.7	1062	2.5	1.1	0.6-1.9
. ≥4	706	2.5	1.0	0.5-1.9	898	3.1	1.2	0.7-2.0
								
Body mass index								
Normal (18.5-24.9)	979	1.8	1		1509	2.4	1	
Underweight (< 18.5)	13	0	-		45	1.4	0.6	0.1-4.6
Overweight (25.0-29.9)	1245	3.1	1.6	0.9-2.9	1020	2.9	1.0	0.6-1.7
Obese (≥ 30.0)	457	3.4	1.7	0.8-3.6	533	3.7	1.2	0.6-2.3
								
Waist circumference								
Men: < 94.0 cm, Women: < 80 cm	1066	1.7	1		929	1.5	1	
Men: 94.0-101.9 cm	810	3.8	2.0	1.1-3.5	811	3.0	1.6	0.8-3.5
Women: 80.0-87.9 cm								
Men: ≥ 102.0 cm	943	2.9	1.4	0.8-2.4	1581	3.4	1.5	0.7-2.9
Women: ≥ 88.0 cm								
								
Waist-to-hip ratio^1^								
Normal	328	1.0	1		614	2.5	1	
Increased	1607	2.8	2.4	0.7-8.0	1875	2.3	0.7	0.4-1.4
High	884	3.1	2.4	0.7-8.4	831	3.8	0.9	0.5-1.8
								
Metabolic syndrome^2^								
No	1970	2.4	1		2296	2.7	1	
Yes	847	3.4	1.2	0.7-1.9	1037	2.9	0.7	0.4-1.1
								
Insulin resistance^3^								
Mean, per each standard deviation increase	2821		1.0	0.9-1.1	3348		0.7	0.4-1.3
								
Diabetes								
No	2666	2.5	1		3168	2.7	1	
Type 1	22	9.4	4.7	1.1-20.3	13	0	-	
Type 2	143	5.6	1.6	0.7-3.5	175	4.2	0.7	0.3-1.8
								
C-reactive protein								
Low < 3 mg/L	2362	2.8	1		2722	2.5	1	
High > 3 mg/L	441	2.6	0.8	0.4-1.6	589	3.9	1.3	0.8-2.2
								
Carotid intima-media thickness								
Mean IMT, per each standard deviation (0.23 mm) increase	604		0.7	0.4-1.2	731		0.7	0.3 -1.3

Odds ratios were adjusted for age, education and physical work load factors (working with hands above the shoulder level for at least one hour per day, manual handling of loads heavier than 5 kg at least two times per minute for a minimum of two hours daily and manual handling of loads heavier than 20 kg for at least 10 times per day, working with a vibrating tool for at least two hours, work demanding high handgrip forces for at least one hour, and repetitive movements of the hands or wrists for at least two hours).

^1 ^In men: Normal < 0.9, increased 0.9-1.0 and obese > 1.0. In women: Normal < 0.8, increased 0.8-0.9 and obese > 0.9.

^2 ^Three of the following criteria present: 1) Central obesity, defined as waist circumference > 102 cm in men and > 88 cm in women; 2) triglycerides > 1.7 mmol/l; 3) HDL < 1.0 mmol/l in men and < 1.3 mmol/l in women; 4) systolic blood pressure > 130 mm Hg or diastolic blood pressure ≥ 85 mm Hg; and 5) fasting glucose ≥ 6.1 mmol/l.

^3 ^HOMA-IR: Serum insulin pmol/l × glucose mmol/l/22.5

## Discussion

Our main findings were that weight-related factors, especially abdominal obesity, are associated with shoulder pain in both genders. Abdominal obesity was also associated with chronic rotator cuff tendinitis.

Type 1 diabetes mellitus was associated with chronic rotator cuff tendinitis in men but not in women. We found no consistent associations between other lifestyle and metabolic factors and shoulder pain or chronic rotator cuff tendinitis.

Although there was an association between shoulder pain and all weight-related factors in both men and women, the associations were strongest for waist circumference and waist-to-hip ratio in both men and women. These two measures showed a similar tendency also with regard to rotator cuff tendinitis, suggesting abdominal obesity as an underlying factor. Previous studies have used mainly BMI as the measure of obesity, and the finding of stronger association for abdominal obesity than for obesity measured with BMI is novel. Our results suggest that waist and hip circumference are better measures of obesity than BMI for future studies on shoulder disorders.

We found an association between type 1 diabetes mellitus and rotator cuff tendinitis in men. Men and women with type 1 diabetes mellitus had also higher prevalence of bilateral shoulder pain. In type 1 diabetes mellitus, metabolic by-products of non-enzymic glycation accumulate in tendons, and shoulder pain and other tendon problems are well known to clinicians treating diabetic patients [21].

Also type 2 diabetes mellitus, the metabolic syndrome and carotid IMT were associated with shoulder pain in men. Men with type 2 diabetes mellitus had also higher prevalence of the rotator cuff tendinitis. These results suggest that the metabolic syndrome and related indicators of disturbed glucose metabolism play a role in shoulder pain and rotator cuff tendinitis.

Heavy smoking or smoking of long duration was associated with unilateral shoulder pain in men and with bilateral shoulder pain in women. Contradictory findings have been reported on the association between smoking and shoulder pain, and most associations have been found in occupational populations [8]. Other sociocultural factors associated with smoking may lie behind this association. In the present study there was no dose-response relation between smoking and shoulder pain.

Obesity and smoking increase the expression of proinflammatory cytokines, including IL-1, IL-6 and TNFα. Moreover, obesity affects the synthesis of adipokines (e.g. leptin, adiponectin and resistin), which operate in a variety of metabolic and immunologic activities, for instance regulating food intake, insulin resistance and inflammation. Shoulder disorders have been linked with IL-1 [22-24] and IL-6 and TNFα [25-27]. In this respect, raised circulating IL-1, IL-6 and TNFα may aggravate shoulder complaints for example by inducing pain hypersensitivity [28-30] and maintaining inflammation. Previous reports suggest adipokines being involved in articular degenerative disorders [31-33]. Obesity stimulates CRP synthesis mainly due to circulating levels of IL-6 [34]. CRP is involved in inflammatory processes in endothelial cells [35]. Therefore, lifestyle including overeating or smoking and physical inactivity increases the production of proinflammatory cytokines, adipokines and CRP. Obesity, smoking, diabetes mellitus, metabolic syndrome and high C-reactive protein are all risk factors of atherosclerosis.

Many pathological conditions may cause shoulder pain. Of clinically defined disorders, rotator cuff tendinitis is the most prevalent, whereas clinically manifest osteoarthritis is rare in the shoulder [36,37]. As a joint with high range of motion and a long lever arm, the shoulder is liable to injuries that may heal only partially and leave residual symptoms.

The associations of smoking and obesity with shoulder pain were stronger for unilateral pain in men and for bilateral pain in women. Atherosclerosis and disturbed glucose metabolism affect the nutrition of the shoulder structures and may render them liable to injuries from physical loads and traumas and impair healing. Men are exposed to higher physical loads at work and may participate in physically more demanding leisure activities and thereby be at a higher risk of shoulder injuries. In women, the associations of lifestyle and metabolic factors with bilateral shoulder pain suggest systemic underlying mechanisms. In men other exposures, such as traumatic injury, superimposed on vascular susceptibility can trigger a unilateral clinical manifestation.

The limitation of the present study is its cross sectional nature. However, the nature of some of the predictors can help us understand the types of the found associations. Obesity, smoking and increased carotid IMT are relatively long lasting conditions. Therefore, the presence of these factors before the onset of shoulder pain is likely and they could be true predictors of shoulder pain and disorders, and a reverse causal association is less likely.

The diagnosis of chronic rotator cuff tendinitis was set with predefined criteria after a standardized physical examination. The categorization into possible or definite was based on physician subjective assessment. Since our sample was a population cross section, most cases were at a less acute stage for which clinical diagnostic criteria may be difficult to apply. In order to increase sensitivity we considered also possible cases in our case definition. Even though we had a large population sample, the study had low power for some of cardiovascular risk factors, e.g., diabetes mellitus.

## Conclusions

Metabolic factors, especially abdominal obesity, and carotid intima-media thickness were associated with shoulder pain. Type 1 diabetes mellitus and abdominal obesity were associated with chronic rotator cuff tendinitis in men. Atherosclerosis and disturbed glucose metabolism may be the underlying mechanisms, although not fully supported by the findings of this study. In addition to metabolic factors, mechanical factors, such as injuries or physical work load exposures, may play a role. This is suggested by stronger associations of smoking, abdominal obesity and metabolic syndrome with unilateral than bilateral shoulder pain in men. Prospective studies are needed to further investigate the role of lifestyle and metabolic factors in shoulder disorders.

## Competing interests

The authors declare that they have no competing interests.

## Authors' contributions

MR participated in the design of the study and drafted the manuscript. RS participated in the design of the study, manuscript drafting and performed the statistical analyses. JK, AJ and MH participated in the design of the study and critically reviewed the manuscript. EV-J launched and coordinated the study, and participated in its design and manuscript drafting. All authors read and approved the final manuscript.

## Pre-publication history

The pre-publication history for this paper can be accessed here:

http://www.biomedcentral.com/1471-2474/11/165/prepub

## Supplementary Material

Additional file 1 **Appendices**. Appendix 1: Gender-specific univariable odds ratios of unilateral or bilateral shoulder pain by lifestyle and metabolic factors, Health 2000 Survey, 2000-2001. Appendix 2: Gender-specific univariable odds ratios of chronic rotator cuff tendinitis by lifestyle and metabolic factors, Health 2000 Survey, 2000-2001. Click here for file
