# Supplementary material for: Lifestyle and metabolic factors in relation to shoulder pain and rotator cuff tendinitis: A population-based study
Source: BMC Musculoskelet Disord. 2010 Jul 20;11:165. doi: 10.1186/1471-2474-11-165 (PMC3161397; doi:10.1186/1471-2474-11-165)
Supplement: Additional file 1 — Appendices. Appendix 1: Gender-specific univariable odds ratios of unilateral or bilateral shoulder pain by lifestyle and metabolic factors, Health 2000 Survey, 2000-2001. Appendix 2: Gender-specific univariable odds ratios of chronic rotator cuff tendinitis by lifestyle and metabolic factors, Health 2000 Survey, 2000-2001. [file 1471-2474-11-165-S1.DOC]

**Appendices**

**Appendix 1: Gender-specific univariable odds ratios of unilateral or bilateral shoulder pain by lifestyle and metabolic factors, Health 2000 Survey, 2000-2001**

| Characteristic | Men | | | | |  | Women | | | | |
| --- | --- | --- | --- | --- | --- | --- | --- | --- | --- | --- | --- |
|  | Unilateral | |  | Bilateral | |  | Unilateral | |  | Bilateral | |
|  | OR | 95% CI |  | OR | 95% CI |  | OR | 95% CI |  | OR | 95% CI |
|  |  |  |  |  |  |  |  |  |  |  |  |
| Age, 1-year increase | 1.02 | 1.01-1.03 |  | 1.04 | 1.03-1.06 |  | 1.03 | 1.02-1.04 |  | 1.05 | 1.04-1.06 |
| Year of education, 1-year increase | 0.93 | 0.90-0.97 |  | 0.82 | 0.78-0.86 |  | 0.90 | 0.87-0.93 |  | 0.82 | 0.78-0.86 |
|  |  |  |  |  |  |  |  |  |  |  |  |
| Smoking status |  |  |  |  |  |  |  |  |  |  |  |
| Never smoker | 1 |  |  | 1 |  |  | 1 |  |  | 1 |  |
| Former smoker | 1.6 | 1.1-2.4 |  | 1.3 | 0.8-1.9 |  | 0.8 | 0.6-1.1 |  | 0.7 | 0.5-1.1 |
| Occasional smoker | 1.2 | 0.6-2.2 |  | 0.5 | 0.2-1.3 |  | 0.6 | 0.3-1.2 |  | 0.1 | 0.0-0.6 |
| Current smoker |  |  |  |  |  |  |  |  |  |  |  |
| <10 pack-years | 1.6 | 1.0-2.6 |  | 0.9 | 0.5-1.7 |  | 0.8 | 0.4-1.3 |  | 0.4 | 0.2-0.8 |
| 10-20 pack-years | 1.1 | 0.5-2.3 |  | 0.4 | 0.1-1.6 |  | 0.6 | 0.3-1.2 |  | 1.0 | 0.5-1.7 |
| >20 pack-years | 2.2 | 1.4-3.3 |  | 0.8 | 0.4-1.6 |  | 0.5 | 0.2-0.9 |  | 0.4 | 0.2-0.9 |
|  |  |  |  |  |  |  |  |  |  |  |  |
| Exercise (times/week) |  |  |  |  |  |  |  |  |  |  |  |
|   1 | 1 |  |  | 1 |  |  | 1 |  |  | 1 |  |
| 2–3 | 1.3 | 0.9-1.7 |  | 0.9 | 0.6-1.4 |  | 1.1 | 0.8-1.4 |  | 0.7 | 0.5-1.1 |
|  > 4 | 0.8 | 0.5-1.1 |  | 1.2 | 0.7-1.9 |  | 1.0 | 0.7-1.3 |  | 0.8 | 0.5-1.2 |
|  |  |  |  |  |  |  |  |  |  |  |  |
| Body mass index |  |  |  |  |  |  |  |  |  |  |  |
| Normal (18.5-24.9) | 1 |  |  | 1 |  |  | 1 |  |  | 1 |  |
| Underweight (<18.5) | - |  |  | - |  |  | 0.4 | 0.1-1.9 |  | 0.4 | 0.1-3.1 |
| Overweight (25.0-29.9) | 1.3 | 0.9-1.7 |  | 1.5 | 1.0-2.3 |  | 1.5 | 1.1-1.9 |  | 1.5 | 1.0-2.0 |
| Obese (>30.0) | 1.6 | 1.1-2.3 |  | 1.6 | 1.0-2.5 |  | 1.6 | 1.1-2.3 |  | 2.2 | 1.5-3.4 |
|  |  |  |  |  |  |  |  |  |  |  |  |
| Waist circumference |  |  |  |  |  |  |  |  |  |  |  |
| Men: < 94.0 cm,  Women: <80 cm | 1 |  |  | 1 |  |  | 1 |  |  | 1 |  |
| Men: 94.0-101.9 cm  Women: 80.0-87.9 cm | 1.9 | 1.3-2.7 |  | 1.3 | 0.8-1.9 |  | 1.7 | 1.3-2.4 |  | 2.4 | 1.5-3.9 |
| Men: >102.0 cm  Women: >88.0 cm | 2.0 | 1.5-2.8 |  | 1.5 | 1.1-2.2 |  | 2.0 | 1.5-2.7 |  | 4.2 | 2.8-6.4 |
|  |  |  |  |  |  |  |  |  |  |  |  |
| Waist-to-hip ratio^1^ |  |  |  |  |  |  |  |  |  |  |  |
| Normal | 1 |  |  | 1 |  |  | 1 |  |  | 1 |  |
| Increased | 2.4 | 1.4-4.1 |  | 3.6 | 1.5-8.4 |  | 1.1 | 0.8-1.5 |  | 1.5 | 0.9-2.4 |
| High | 3.0 | 1.7-5.3 |  | 4.1 | 1.7-9.5 |  | 1.6 | 1.1-2.4 |  | 3.6 | 2.2-5.9 |
|  |  |  |  |  |  |  |  |  |  |  |  |
| Metabolic syndrome^2^ |  |  |  |  |  |  |  |  |  |  |  |
| No | 1 |  |  | 1 |  |  | 1 |  |  | 1 |  |
| Yes | 1.7 | 1.3-2.1 |  | 1.3 | 0.9-1.8 |  | 1.2 | 0.9-1.6 |  | 2.1 | 1.6-2.8 |
|  |  |  |  |  |  |  |  |  |  |  |  |
| Insulin resistance^3^ |  |  |  |  |  |  |  |  |  |  |  |
| Mean, per each standard deviation increase | 1.0 | 0.9-1.1 |  | 1.0 | 0.9-1.1 |  | 1.4 | 1.1-1.6 |  | 1.4 | 1.2-1.7 |
|  |  |  |  |  |  |  |  |  |  |  |  |
| Diabetes |  |  |  |  |  |  |  |  |  |  |  |
| No | 1 |  |  | 1 |  |  | 1 |  |  | 1 |  |
| Type 1 | - | - |  | 1.8 | 0.4-8.1 |  | - | - |  | 2.3 | 0.6-9.2 |
| Type 2 | 2.2 | 1.3-3.5 |  | 3.1 | 1.7-5.8 |  | 2.1 | 1.4-3.1 |  | 2.5 | 1.5-4.0 |
|  |  |  |  |  |  |  |  |  |  |  |  |
| C-reactive protein (High >3 mg/L vs. low <3 mg/L) | 1.4 | 0.9-2.0 |  | 1.3 | 0.8-2.1 |  | 1.2 | 0.9-1.6 |  | 1.8 | 1.3-2.5 |
|  |  |  |  |  |  |  |  |  |  |  |  |
| Mean IMT, per each standard deviation (0.23 mm) increase | 1.1 | 0.8-1.5 |  | 1.4 | 1.1-1.8 |  | 0.8 | 0.6-1.1 |  | 1.2 | 0.9-1.6 |
|  |  |  |  |  |  |  |  |  |  |  |  |
| **Physical load factors** |  |  |  |  |  |  |  |  |  |  |  |
| Working with hands above the shoulder level | 1.6 | 1.2-2.2 |  | 2.5 | 1.8-3.5 |  | 1.8 | 1.4-2.4 |  | 3.2 | 2.4-4.2 |
| Manual handling of loads > 5 kg | 1.3 | 0.9-1.7 |  | 3.4 | 2.4-4.7 |  | 1.9 | 1.5-2.5 |  | 2.8 | 2.1-3.8 |
| Manual handling of loads > 20 kg | 1.6 | 1.2-2.1 |  | 2.7 | 2.0-3.6 |  | 1.8 | 1.4-2.4 |  | 2.5 | 1.9-3.4 |
| Using vibrating tools | 1.4 | 1.0-2.0 |  | 2.9 | 2.0-4.2 |  | 2.2 | 1.2-3.8 |  | 3.9 | 2.2-6.6 |
| High handgrip forces | 1.4 | 1.0-1.8 |  | 2.9 | 2.0-4.1 |  | 1.9 | 1.5-2.5 |  | 2.9 | 2.1-3.9 |
| Repetitive movements of the hands or wrists | 1.3 | 0.9-1.7 |  | 1.7 | 1.2-2.3 |  | 1.7 | 1.3-2.1 |  | 2.3 | 1.7-3.1 |
|  |  |  |  |  |  |  |  |  |  |  |  |

^1^ In men: Normal <0.9, Increased 0.9-1.0 and High >1.0. In women: Normal <0.8, Increased 0.8-0.9 and High >0.9.

^2^ Three of the following criteria present: 1) Central obesity, defined as waist circumference > 102 cm in men and > 88 cm in women; 2) triglycerides > 1.7 mmol/l; 3) HDL < 1.0 mmol/l in men and <1.3 mmol/l in women; 4) systolic blood pressure > 130 mm Hg or diastolic blood pressure > 85 mm Hg; and 5) fasting glucose > 6.1 mmol/l.

^3^ HOMA-IR: Serum insulin pmol/l x glucose mmol/l /22.5

**Appendix 2: Gender-specific univariable odds ratios of chronic rotator cuff tendinitis by lifestyle and metabolic factors, Health 2000 Survey, 2000-2001**

| Characteristic | Men | |  | Women | |
| --- | --- | --- | --- | --- | --- |
|  | OR | 95% CI |  | OR | 95% CI |
|  |  |  |  |  |  |
| Age, 1-year increase | 1.03 | 1.01-1.05 |  | 1.03 | 1.01-1.04 |
| Year of education, 1-year increase | 0.93 | 0.87-0.99 |  | 0.92 | 0.86-0.97 |
|  |  |  |  |  |  |
| Smoking status |  |  |  |  |  |
| Never smoker | 1 |  |  | 1 |  |
| Former smoker | 1.5 | 0.8-2.7 |  | 0.9 | 0.5-1.5 |
| Occasional smoker | 0.5 | 0.1-2.1 |  | 0.2 | 0.1-1.3 |
| Current smoker |  |  |  |  |  |
| <10 pack-years | 0.3 | 0.1-1.5 |  | 0.7 | 0.2-1.7 |
| 10-20 pack-years | 0.3 | 0.1-2.1 |  | 1.0 | 0.4-2.4 |
| >20 pack-years | 1.5 | 0.6-3.5 |  | 0.3 | 0.1-1.4 |
|  |  |  |  |  |  |
| Exercise (times/week) |  |  |  |  |  |
|   1 | 1 |  |  | 1 |  |
| 2–3 | 1.5 | 0.9-2.6 |  | 1.0 | 0.6-1.7 |
|   4 | 1.1 | 0.5-2.3 |  | 1.2 | 0.7-2.0 |
|  |  |  |  |  |  |
| Body mass index |  |  |  |  |  |
| Normal (18.5-24.9) | 1 |  |  | 1 |  |
| Underweight (<18.5) | - |  |  | 0.6 | 0.1-4.4 |
| Overweight (25.0-29.9) | 1.7 | 0.9-3.0 |  | 1.2 | 0.7-2.0 |
| Obese (>30.0) | 1.9 | 0.9-3.9 |  | 1.5 | 0.8-2.8 |
|  |  |  |  |  |  |
| Waist circumference |  |  |  |  |  |
| Men: < 94.0 cm,  Women: <80 cm | 1 |  |  | 1 |  |
| Men: 94.0-101.9 cm  Women: 80.0-87.9 cm | 2.2 | 1.2-4.0 |  | 2.0 | 0.9-4.1 |
| Men: >102.0 cm  Women: >88.0 cm | 1.7 | 0.9-3.0 |  | 2.3 | 1.2-4.3 |
|  |  |  |  |  |  |
| Waist-to-hip ratio^1^ |  |  |  |  |  |
| Normal | 1 |  |  | 1 |  |
| Increased | 3.0 | 0.9-9.8 |  | 0.9 | 0.5-1.7 |
| High | 3.4 | 1.0-11.4 |  | 1.5 | 0.8-2.9 |
|  |  |  |  |  |  |
| Metabolic syndrome^2^ |  |  |  |  |  |
| No | 1 |  |  | 1 |  |
| Yes | 1.4 | 0.8-2.2 |  | 1.1 | 0.7-1.6 |
|  |  |  |  |  |  |
| Insulin resistance^3^ |  |  |  |  |  |
| Mean, per each standard deviation increase | 1.0 | 0.9-1.1 |  | 1.0 | 0.8-1.3 |
|  |  |  |  |  |  |
| Diabetes |  |  |  |  |  |
| No | 1 |  |  | 1 |  |
| Type 1 | 4.1 | 0.98-16.8 |  | - |  |
| Type 2 | 2.3 | 1.1-4.8 |  | 1.6 | 0.7-3.3 |
|  |  |  |  |  |  |
| C-reactive protein (High >3 mg/L vs. low <3 mg/L) | 1.0 | 0.5-1.8 |  | 1.5 | 0.9-2.5 |
|  |  |  |  |  |  |
| Mean IMT, per each standard deviation (0.23 mm) increase | 0.7 | 0.4-1.3 |  | 0.7 | 0.3-1.3 |
|  |  |  |  |  |  |
| **Physical load factors** |  |  |  |  |  |
| Working with hands above the shoulder level | 1.5 | 0.9-2.3 |  | 2.0 | 1.3-3.1 |
| Manual handling of loads > 5 kg | 1.2 | 0.7-2.0 |  | 1.8 | 1.1-2.9 |
| Manual handling of loads > 20 kg | 1.4 | 0.8-2.2 |  | 2.6 | 1.6-4.0 |
| Using vibrating tools | 1.1 | 0.6-1.9 |  | 2.4 | 1.1-5.5 |
| High handgrip forces | 1.6 | 0.9-2.6 |  | 1.9 | 1.2-3.0 |
| Repetitive movements of the hands or wrists | 1.6 | 0.9-2.5 |  | 1.2 | 0.7-1.9 |
|  |  |  |  |  |  |

^1^ In men: Normal <0.9, Increased 0.9-1.0 and High >1.0. In women: Normal <0.8, Increased 0.8-0.9 and High >0.9.

^2^ Three of the following criteria present: 1) Central obesity, defined as waist circumference > 102 cm in men and > 88 cm in women; 2) triglycerides > 1.7 mmol/l; 3) HDL < 1.0 mmol/l in men and<1.3 mmol/l in women; 4) systolic blood pressure > 130 mm Hg or diastolic blood pressure > 85 mm Hg; and 5) fasting glucose > 6.1 mmol/l.

^3^ HOMA-IR: Serum insulin pmol/l x glucose mmol/l /22.5
